# Reevaluation of risk factors for time to subsequent events after first stroke occurrence using a new weighted all-cause effect measure

**DOI:** 10.1186/s12889-020-08971-4

**Published:** 2020-06-01

**Authors:** Ann-Kathrin Ozga, Bernhard Rauch, Frederick Palm, Christian Urbanek, Armin Grau, Heiko Becher, Geraldine Rauch

**Affiliations:** 1 Institute of Medical Biometry and Epidemiology, University Medical Center Hamburg-Eppendorf, Martinistraße 52, 20246 Hamburg, Germany; 2 IHF GmbH, Institut für Herzinfarktforschung, Bremserstraße 79, 67063 Ludwigshafen, Germany; 3 Helios Klinikum Schleswig, St. Jürgener Straße, 1-3, 24837 Schleswig, Germany; 4 Department of Neurology, Städtisches Klinikum Ludwigshafen am Rhein, University of Heidelberg, Bremserstraße 79, 67063 Ludwigshafen, Germany; 5 Charité - Universitätsmedizin Berlin, corporate member of Freie Universität Berlin, Humboldt-Universität zu Berlin, Institute of Biometry and Clinical Epidemiology, Charitéplatz 1, 10117 Berlin, Germany; 6 Berlin Institute of Health (BIH), Anna-Louisa-Karsch 2, 10178 Berlin, Germany; 7 Institute of Global Health, University Hospital Heidelberg, Im Neuenheimer Feld 130/3, 69120 Heidelberg, Germany

**Keywords:** Stroke, Death, Composite endpoint, Time-to-event, Risk factors, Weighted all-cause hazard ratio

## Abstract

**Background:**

Risk diseases and risk factors for stroke include atrial fibrillation, hypertension, diabetes mellitus, smoking, and elevated LDL-cholesterol. Due to modern treatment options, the impact of these risk diseases on subsequent cardiovascular events or death after a first stroke is less clear and needs to be elucidated. We therefore aimed to get insights into the persistence of adverse prognostic effects of these risk diseases and risk factors on subsequent stroke or death events 1 year after the first stroke by using the new weighted all-cause hazard ratio.

**Methods:**

This study evaluates the 1 year follow-up of 470 first ever stroke cases identified in the area of Ludwigshafen, Germany, with 23 deaths and 34 subsequent stroke events. For this purpose, the recently introduced “weighted all-cause hazard ratio” was used, which allows a weighting of the competing endpoints within a composite endpoint. Moreover, we extended this approach to allow an adjustment for covariates.

**Results:**

None of these risk factors and risk diseases, most probably being treated after the first stroke, remained to be associated with a subsequent death or stroke [weighted hazard ratios (95% confidence interval) for diabetes mellitus, atrial fibrillation, high cholesterol, hypertension, and smoking are 0.4 (0.2–0.9), 0.8 (0.4–2.2), 1.3 (0.5–2.5), 1.2 (0.3–2.7), 1.6 (0.8–3.6), respectively]. However, when analyzed separately in terms of death and stroke, the risk factors and risk diseases under investigation affect the subsequent event rate to a variable degree.

**Conclusions:**

Using the new weighted hazard ratio, established risk factors and risk diseases for the occurrence of a first stroke do not remain to be significant predictors for subsequent events like death or recurrent stroke. It has been demonstrated that the new weighted hazard ratio can be used for a more adequate analysis of cardiovascular risk and disease progress. The results have to be confirmed within a larger study with more events.

## Background

Stroke is the second leading cause of death worldwide [1, 2]. Studies showed a risk of dying 1 year after a first stroke of around 40% [3, 4]. Commonly known risk factors and risk diseases for a first ever stroke like atrial fibrillation, hypertension, diabetes mellitus, smoking, or high cholesterol are well accepted of being responsible for about 50–70% of all strokes [5, 6]. Risk factors for subsequent strokes after a first non-fatal stroke event were also analyzed but more heterogeneous results were gained with indicators including diabetes mellitus, coronary artery disease, hypertension, a high baseline score on the “National Institute of Health Stroke Scale”, intracranial arterial stenosis, or hyperlipidemia [7–9]. Although a recent meta-analysis suggested that hypertension, diabetes mellitus, atrial fibrillation, and coronary heart disease might be risk factors for a subsequent stroke occurrence, they did not consider a time-to-event analysis [10]. Even less analyzed are risk factors for the “time to death” after a first ever stroke, which might differ from the common risk factors and risk diseases for the “time to first ever stroke”. Although the rate of dying as a direct consequence of a stroke was reported to be high, it appeared to be difficult to quantify additional risk factors and risk diseases that influence the disease progress after a first stroke [3, 4]. Heterogeneous groups of patients may be one reason for this observation, as different stroke etiologies markedly influence the risk of subsequent events. Moreover, the consequent implementation of secondary prevention strategies - especially protective medication - markedly reduces the rate of subsequent clinical events [11]. Therefore, patients at risk in secondary prevention after a first non-fatal stroke represent a special selection and need to be newly defined in general, but also in all-day care. Finally, there are extended efforts in medical research to prevent subsequent strokes after a first stroke, thereby continuously changing risk patterns in secondary prevention [12].

In the present study, we aim to deliver a reevaluation of potential risk factors and risk diseases for premature death or stroke after a first stroke event. Taking into account that death acts as a competing event to a subsequent non-fatal stroke, the composite endpoint with the two components “time to subsequent death” or “time to subsequent stroke” was taken as primary endpoint. These two endpoints are clearly of different clinical relevance, as a fatal event always represents the worst outcome. This has to be addressed in the analysis of composite endpoints. However, common statistical methods for composite endpoints are usually based on the all-cause hazard ratio, which ignores the different impact of the components. To overcome this problem, we recently introduced the weighted all-cause hazard ratio to simplify interpretation of a composite endpoint with competing components of different clinical relevance [13, 14]. In the present study, this new methodologic approach was extended for a situation to incorporate an adjustment for confounders. This allows to adequately model a complex time-to-event framework, in which two competing events with different clinical relevance are of primary interest.

The primary aim of this exploratory study was to (re-)evaluate the effect of common risk factors and risk diseases for a first stroke on the “time to subsequent death or stroke” using a new weighted effect measure. As only 60% of patients survive more than 1 year after a first stroke, it is of high clinical relevance to estimate and characterize as exactly as possible the ongoing risk under actual treatment conditions [3, 4]. We additionally aimed to illustrate how the weighted all-cause hazard ratio helps to more adequately describe the progress of risk along a chronic disease under contemporary treatment, a method of potential interest in public health.

## Methods

### Study population

**Fig. 1:**
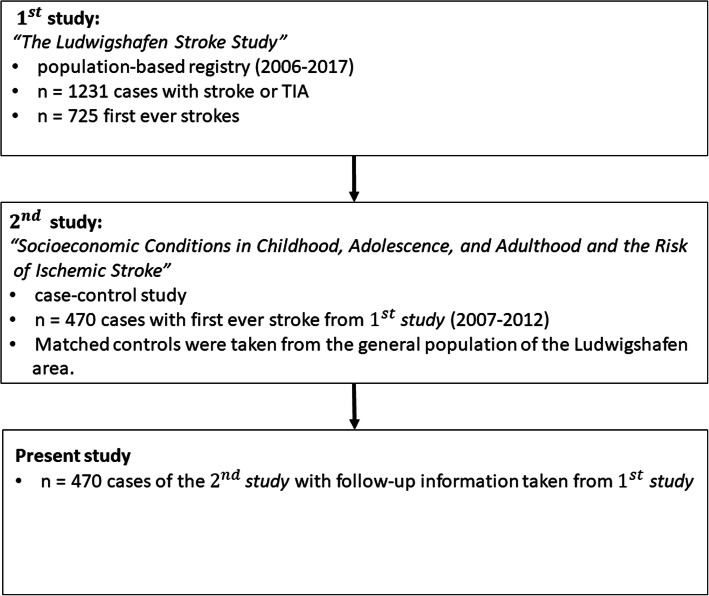
Flow chart describing basis of study sample

This is a study on *n* = 470 incident cases with ischemic stroke with a 1-year follow-up. These cases are taken from a recently published case-control study [15] which was embedded in the Ludwigshafen population-based stroke registry covering about 93% of all stroke patients below the age of 80 years in the urbanized industrial area of Ludwigshafen [16]. Figure 1 shows the corresponding flow-chart.

The diagnosis of “stroke” is based on the definition of the World Health Organization [17].

Cases gave written informed consent. They were of white ethnicity and between 18 and 80 years of age. Exclusion criteria were additional previous events like stroke of any etiology, acute transient ischemic attack, intracerebral, subdural or subarachnoidal hemorrhage, myocardial infarction within the previous 90 days, dementia, severe aphasia, and other relevant communication barriers as well as withdrawal of consent.

These cases, i.e. the underlying study population analyzed in this work, underwent a personal interview with trained interviewers using a standardized questionnaire. The interviewers were guided by a handbook. Instructions and trainings were given to each of the 11 interviewers (6 medical doctors, 3 nurses and 4 medical doctor students) prior to study start. The quality of data collection was ensured by regular training and monitoring of interviewers. Data were double entered and checked for completeness and plausibility.

Sociodemographic data and behavioral factors were self-reported, whereas cardio-metabolic data were checked by medical personnel using medical records (F.P.). Also, based on medical records, previous risk diseases were assessed using standard definitions for hypertension, diabetes mellitus, hypercholesterolemia, and atrial fibrillation. Body weight and body height were both measured and assessed by the questionnaire.

Active follow-up was done by phone after one, three and 12 months following the diagnosis. These contacts exclusively served to assess the occurrence of a subsequent stroke during the time period since the previous visit. Therefore, no exact event time points were given for the event “subsequent stroke”. Vital status was obtained from the local population registry and date of death was assessed for deceased cases. Neither the severity nor the kind of second stroke were assessed by this follow-up.

### Statistical methods

Baseline characteristics of patients are summarized with descriptive statistical methods. Continuous variables are described by means and standard deviations. Categorical data are summarized as absolute and relative frequencies.

To analyze the risk factors that might affect the time to death or subsequent stroke we applied the weighted all-cause hazard ratio, thereby taking into account the occurrence of competing events [13, 14]. Thus, different “relevance weights” for the two competing components “death” and “subsequent stroke” were applied. For choosing the weights, we followed the recommendations for finding a weighting scheme as described by Ozga & Rauch, which are also explained in the Additional file 1.

As a first step, we determined “death” to be the most relevant outcome, and thus a weight of “1” was assigned for “death”. In a second step, the event “subsequent stroke” was set into relation to “death” by answering the purely theoretical question of “how many strokes are considered to be as harmful as one death?”. The clinical consideration that “stroke” represents a severe clinical event but may be survived, thereby resulting in more “stroke events” than “deaths” within the observed time period, led us to choose a weight of 0.7 for the event “stroke”. Additionally, the “disability weights” as described in the “Global Burden of Disease” study 2016 [18] supported our decision. Although the definitions of these “disability weights” are not directly comparable to our approach (i.e. weights were chosen on the basis of medical records like speech impairment, or being confined to bed or wheelchair), they also set the event “stroke” in relation to “death”. Disability weights for ischemic stroke ranged from 0.019 to 0.588 with a maximal upper confidence limit of 0.744 [18].

The weighted all-cause hazard ratio was originally introduced for a two-group comparison. For our analysis we extended this effect measure to adjust for confounders (see Additional file 1). In addition to the weighted all-cause hazard ratio, we also provide results for the non-weighted all-cause hazard ratio resulting from the Cox proportional hazards model and for the cause-specific hazard ratios for each component separately. Along with the analysis using other weighting schemes, these later analyses are interpreted as sensitivity analyses. All hazard ratios are reported with 95%-confidence intervals. For the weighted all-cause hazard ratio, these are estimated via bootstrap sampling with 10,000 runs. Risk diseases and risk factors evaluated at baseline were atrial fibrillation, hypertension, diabetes mellitus, hypercholesterolemia and smoking. Furthermore, all estimates were adjusted for age (continuous) and sex.

The stroke risk factors considered in this study were defined as follows (unpublished study report):

- *atrial fibrillation*: “persistent” and “paroxysmal”
- *hypertension*: values ≥ 140/90 mmHg at rest at three time points
- *diabetes mellitus*: fasting-plasma-glucose > 125 mg/dl (> 7 mmol/l) or peak blood sugar > 200 mg/dl (> 11 mmol/l) or two hours value with oral test for glucose tolerance > 200 mkg/dl (> 11 mmol/l)
- *smoking*: consumption of either a minimum of one cigarette/day, five cigarettes/week, one pack of cigarettes/month, two cigars or two pipes/week over a period of 6 month or more during life
- *hypercholesterolemia*: fasting total serum cholesterol ≥ 200 mg/dl

Since no information was available for any treatment regarding vascular risk factors and risk diseases, the therapeutic regimen could not be included as a predictor in the analysis.

As mentioned above, the exact time of an event has only been delivered for deaths but not for non-fatal strokes. In the latter cases only time intervals were presented (“interval censoring”). For unadjusted univariable analyses, some methods were proposed to account for this, such as different score tests or a parametric model [19–22]. The parametric model can be extended easily to a model adjusting for confounders. However, since we have no information about the underlying event-time distributions and a misspecification of the distribution can introduce serious bias, we decided against a parametric approach and in favor of a naive approach. If the event time is known to fall in an interval, the naive approach assumes that the event occurred at the upper boundary of the interval, thus at the next time point observed. To assess the robustness of this naive approach, we repeated the analyses with the event times replaced by the lower boundary of the interval and by the middle of the interval. We used the statistical software R Version 3.5.1 for all analyses [23]. The algorithm Mersenne Twister is used for random number generating needed to calculate the bootstrap confidence intervals [24].

## Results

### Characteristics of included patients

Baseline and further data characteristics of the 470 patients under investigation are outlined in Table 1. Physical activity and education level are only given for description of baseline characteristics but are not considered in the analysis of risk factors. Within the follow-up period of 1 year 23 (4.9%) patients died and 34 (7.2%) experienced a subsequent stroke. Thus, 34 event times were interval-censored. The median time-to-event (either “death” or “stroke”) was 3.2 months (1st - 3rd quantile: 1.5–11.5). The median time to death was 4.1 months (1st - 3rd quantile: 2.5–8.7) and the median time to subsequent stroke was 3.0 months (1st - 3rd quantile: 1.1–11.6), respectively. The remaining 413 patients were censored at 12 months plus 35 days (395 days).

**Table 1:** Characteristics of the study population

Characteristics	Total observations n = 470	Without subsequent stroke or death ***n*** = 413 (87.9%)	2nd stroke ***n*** = 34 (7.2%)	Death ***n*** = 23 (4.9%)
**Age** (years) – mean ± SD	66.3 ± 10.9	66.1 ± 10.9	67.5 ± 10.1	68.9 ± 11.2
**Male** - n (%)	282 (60.0)	244 (86.5)	23 (8.2)	15 (5.3)
**Education level**				
Primary/low-secondary school - n (%)	359 (76.4)	313 (87.2)	25 (7.0)	21 (5.8)
Secondary school- n (%)	56 (11.9)	52 (92.9)	3 (5.4)	1 (1.8)
German Abitur/University - n (%)	55 (11.7)	48 (87.3)	6 (10.9)	1 (1.8)
**Body-mass-index** (kg/m^2^) – mean ± SD	28.8 ± 5.2	28.8 ± 5.3	28.1 ± 4.3	29.1 ± 4.1
Physical activity *)				
no or little activity - n (%)	315 (67.7)	278 (88.3)	23 (7.3)	14 (4.4)
1–2 days per week - n (%)	67 (14.4)	57 (85.1)	6 (9.0)	4 (6.0)
3–7 days per week - n (%)	83 (17.8)	73 (88.0)	5 (6.0)	5 (6.0)
**Smoking** - n (%)	145 (30.9)	121 (83.5)	15 (10.3)	9 (6.2)
**Diabetes mellitus** - n (%)	145 (30.9)	132 (91.0)	3 (2.1)	10 (6.9)
**Atrial fibrillation** - n (%)	92 (19.6)	82 (89.1)	4 (4.5)	6 (6.5)
**Hypercholesterolemia** - n (%)	342 (72.8)	297 (86.8)	25 (7.3)	20 (5.9)
**Hypertension** - n (%)	407 (86.6)	358 (88.0)	29 (7.1)	20 (4.9)

*SD* standard deviation; *n* number; *) *n* = 465

### Analysis of the stroke risk factors and risk diseases

**Fig. 2:**
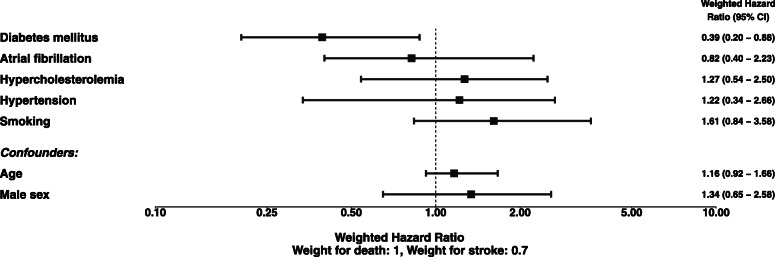
Adjusted weighted hazard ratios with weights “1” for death and “0.7” for stroke. All effects are adjusted for all other independent variables. Confidence intervals were produced via bootstrap sampling. For age the hazard ratio refers to a 10 year’s difference

**Fig. 3:**
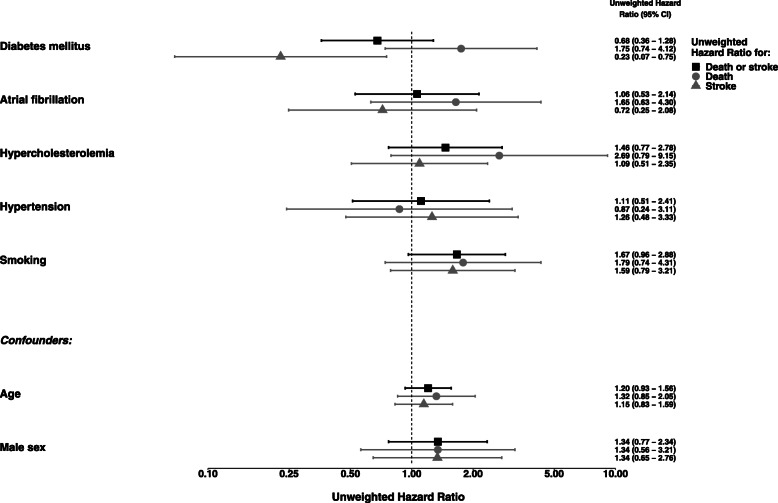
Adjusted unweighted all-cause hazard ratio and cause-specific hazard ratios for sensitivity analysis. All effects are adjusted for all other independent variables. Confidence intervals were produced via bootstrap sampling. For age the hazard ratio is for a 10 year’s difference

Figure 2 shows the adjusted weighted all-cause hazard ratio (HR) at 395 days after the index stroke with 95% bootstrap confidence intervals (CI). Figure 3 depicts the adjusted unweighted all-cause hazard ratios and the adjusted cause-specific hazard ratios for each event type separately. In each model, adjustment has been performed by including all remaining independent variables. In Fig. 2 it can be seen that for diabetic patients the weighted average risk for a subsequent event is significantly reduced with a weighted HR of 0.39 (95% CI 0.20–0.88) whereas none of the other known risk factors for a first ever stroke show a statistically significant association.

Furthermore, when investigating the cause-specific hazard ratios it can be seen in Fig. 3 that the effect of the different event types can point into opposite directions: e.g. for diabetes mellitus, the cause-specific hazard ratio for “death” suggests a higher mortality risk (1.75; 95%-CI = (0.74–4.12)), but for “subsequent stroke” the event risk is virtually lower (cause-specific hazard ratio 0.23; 95%-CI = (0.07–0.75)). This potentially could indicate a shift from “strokes survived” to “stroke associated with death”, which would be of high clinical relevance for further clinical investigations and clarification. For the other covariates no statistically significant effects were observed.

**Fig. 4:**
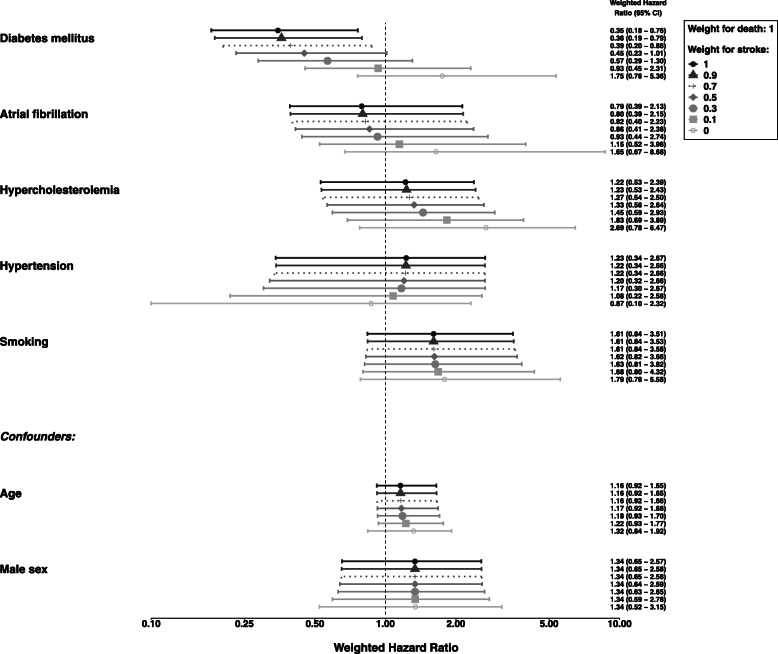
Adjusted weighted hazard ratios with weight “1” for death and weights for stroke ranging between “0” and “1” for sensitivity analysis. All effects are adjusted for all other independent variables. Confidence intervals were calculated via bootstrap sampling. For age the hazard ratio is for a 10 year’s difference

The results for other weighting schemes are outlined in Fig. 4. The differences in the estimated effects between the analyses for the unweighted all-cause effect (Fig. 3) and the analysis with weight “1” for both event types are due to the differences in the estimated baseline hazard: For the weighted approach different cause-specific baseline hazards are assumed, whereas the model for the unweighted estimation implies the same baseline hazard for the included components. The analysis using the lower or mid interval time points for the event type “stroke” delivered similar results (see Additional file 1).

It has to be noted that the weighted composite hazard ratio depends on the cause-specific hazards and thereby also depends on direction and magnitude of the cause-specific hazard ratios. The direction of the weighted composite effect therefore depends on these cause-specific properties and additionally on the weight choice. For larger differences between the cause-specific effects the weighting scheme has the highest impact on the weighted composite hazard ratio.

## Discussion

We presented the association of known major cardiovascular risk factors and risk diseases for a first ever stroke with the combined endpoint “time to death” and “time to second stroke” during a follow-up of 1 year by using a newly developed method for weighted composite endpoints [13, 14]. The presented results show that under the conditions of current treatment and secondary prevention after a first stroke, the rates of death or subsequent non-fatal strokes during the first year of follow-up are not associated anymore with the well accepted and individually ascertained cardiovascular risk factors and risk diseases. These data therefore support the hypothesis that treatment and secondary prevention measures addressing these risk factors and risk diseases are effective in the prevention of subsequent serious clinical events at least during the first year of follow-up.

Notwithstanding, the presented data do not allow to extrapolate these observations to longer time periods, as the actual treatment options may only be effective to postpone secondary clinical events but not to completely prevent them. These events observed during the first year after a first non-fatal stroke therefore need to be evaluated in more detail to get more information on causes and deleterious mechanisms. For example, additional confounders might influence the association seen for diabetic patients, especially with respect to life style and comorbidities. Furthermore, the degree of organ damage also influences the disease progress but could not be explicitly incorporated in the model due to missing information. The time point of starting therapeutic interventions with regard to primary and secondary prevention of stroke also will affect the occurrence of subsequent events during follow-up. Thereby, it is a limitation of this study that the specific treatments including medications could not be included into this evaluation.

The study has some further limitations: The sample size and thus the number of events is small. The mortality in the study group is low due to the selection of “cases” within the underlying case-control study, in which strokes with severe impairment were excluded. However, similar stroke rates were found in other studies [3, 8, 25]. The low event number also results in a limited accuracy of the estimates as expressed by wide confidence intervals. Furthermore, secondary stroke records were based on the patients’ information, which was not verified by medical supervision. The severity of secondary strokes has not been assessed which would have allowed to use individual weights. Additionally, we did not incorporate a possible change of patients’ conditions over time, e.g. if patients stopped smoking. Finally, there was no documentation of the causes of death.

As a methodological result, our analysis emphasizes the importance to take all event types simultaneously into consideration to derive meaningful interpretations. Thereby, the different clinical relevance of competing event types should be taken into account, which can be done with the weighted all-cause hazard ratio yielding a weighted average effect. This methodology considerably strengthens the basis of associations measured in clinical studies and registries due to the consideration of all event times and the length of an individual being at risk for any event. Thus, a disease progress can be more adequately described. Serving as a model for this biometrical approach the present study clearly shows that the associations of well-known risk factors and risk diseases for the occurrence of a first stroke do not apply anymore during the period of secondary prevention. By this new methodologic approach our study differs from other studies, which exclusively examined either the risk for a recurrent stroke or the risk for death [7–9]. However, choosing the appropriate weights remains to be challenging and should be further discussed in detail by medical and statistical experts.

## Conclusion

In summary, we, for the first time, extended the weighted all-cause hazard ratio to adjust for confounders. Thus, it is now generally possible to assess several risk factors by a time-to-event analysis for composite endpoints, thereby incorporating competing endpoints of different clinical relevance. By this way, disease progress can more adequately be described, which is of high relevance for an individualized and clinically successful treatment taking into account the individual risk profiles and comorbidities and thereby allowing a highly individualized therapy as precondition for a successful secondary prevention.

Against this background, our results suggest that most commonly reported risk factors and risk diseases for a first stroke cannot serve as predictors for subsequent stroke events or death anymore. The clinical relevance of our results is limited due to the small number of events and should be verified by testing the presented methodology on the basis of larger populations.

## Supplementary information

**Additional file 1.** The additional file [AdditionalFile.pdf] contains the description of the “Adjusted weighted all-cause hazard ratio” as well as general considerations for choosing the weights, an illustration of correlation between independent variables, and further results for the estimated weighted hazard ratio using the lower or mid interval time points for the event “stroke” and the weights 0.7 for “stroke” and 1 for “death”.

## Data Availability

The datasets used and analyzed during the current study are available from the corresponding author on reasonable request.

## Abbreviations

CI: Confidence interval
HR: Hazard ratio
