# Supplementary material for: Reevaluation of risk factors for time to subsequent events after first stroke occurrence using a new weighted all-cause effect measure
Source: BMC Public Health. 2020 Jun 1;20:817. doi: 10.1186/s12889-020-08971-4 (PMC7268286; doi:10.1186/s12889-020-08971-4)
Supplement: Supplementary file 1 — Additional file 1. The additional file [AdditionalFile.pdf] contains the description of the “Adjusted weighted all-cause hazard ratio” as well as general considerations for choosing the weights, an illustration of correlation between independent variables, and further results for the estimated weighted hazard ratio using the lower or mid interval time points for the event “stroke” and the weights 0.7 for “stroke” and 1 for “death”. [file 12889_2020_8971_MOESM1_ESM.pdf]

Additional File for: ’’Reevaluation of risk factors for time to subsequent events after first stroke occurrence using a new weighted all-cause effect measure’’

Adjusted weighted all-cause hazard ratio

The weighted all-cause hazard ratio was originally introduced for a two group comparison and is time dependent. Assume two different event types, EP_1 and EP_2, and let w_{EP_1} and w_{EP_2} be the pre-defined corresponding relevance weights. The two groups to be compared are denoted as I and C. Then the weighted average hazard ratio is given as:

$$\theta_{CE}^{w}(t) = \frac{w_{EP_1}\lambda_{EP_1}^{I}(t) + w_{EP_2}\lambda_{EP_2}^{I}(t)}{w_{EP_1}\lambda_{EP_1}^{C}(t) + w_{EP_2}\lambda_{EP_2}^{C}(t)}$$

Under equal baseline hazards across the components, it was shown by Ozga and Rauch that this can be reformulated as:

$$\theta_{CE}^{w}(t) = \frac{w_{EP_1}\Lambda_{EP_1}^{I}(t) + w_{EP_2}\Lambda_{EP_2}^{I}(t)}{w_{EP_1}\Lambda_{EP_1}^{C}(t) + w_{EP_2}\Lambda_{EP_2}^{C}(t)}$$

$$= \frac{\sum_{k=1}^{2} w_{EP_k}\Lambda_{EP_k,0}(t)\exp(\beta_{EP_k})}{\sum_{k=1}^{2} w_{EP_k}\Lambda_{EP_k,0}(t)}$$

with the logarithmically transformed cause-specific hazard ratios β_{EP_k}, $k = 1, 2$, which can be estimated via the cause-specific Cox-models. The cumulative baseline hazards $\Lambda_{EP_k,0}(t)$ can be estimated via the cause-specific Nelson-Aalen estimators. Thus, the cumulative baseline hazards are estimated separately for the components and therefore cannot be cancelled.

To additionally adjust for one confounder X_2 (without loss of generality), it is simply possible to plug-in the Cox-estimators of the corresponding multivariable Cox-model:

$$\hat{\theta}_{CE}^{w} = \frac{\sum_{k=1}^{2} w_{EP_k}\hat{\Lambda}_{EP_k,0}(t)\exp(\hat{\beta}_{EP_k} + X_2\hat{\beta}_{EP_k,2})}{\sum_{k=1}^{2} w_{EP_k}\hat{\Lambda}_{EP_k,0}(t)\exp(X_2\hat{\beta}_{EP_k,2})}$$

In the statistic software R the cause-specific cumulative baseline hazards are gained via the *basehaz* function of the survival package applied on the fit of the cause-specific Cox-model (*coxph* function). The cause-specific Cox-models are also used to gain the estimates $\hat{\beta}_{EP_1}, \hat{\beta}_{EP_2}, \hat{\beta}_{EP_1,2}$, and $\hat{\beta}_{EP_2,2}$.

General considerations for choosing the weights

In the following the proposed steps for the choice of weights for the weighted all-cause hazard ratio are listed as originally published by Ozga and Rauch:

1. Identify the clinically most relevant event type (e.g. ’’death’’) and assign a weight of 1.

2. Answer the following question ”How many events of a specific type can be considered as equally harmful than observing one event (or any other amount of reference events) in the clinically most relevant endpoint?”.
3. If there are some assumption about the form of the underlying event time distributions (survival function), graphically investigate the expected survival time by plotting the unweighted and weighted event time distribution for different weighting schemes.

Correlation between independent variables

To illustrate any correlation between variables that might influence the time-to-event outcome, Figure 1 shows the heatmap of correlation coefficients. For two binary variables Pearson's contingency coefficient is given, for the correlation between a binary and a continuous variable the biserial correlation is shown, and for two continuous variables the Pearson correlation coefficient was used. For Pearson's contingency coefficient only non-negative values are possible where a high correlation is near to 1.The other coefficients can take values between -1 and 1 where values near those limits correspond to a high correlation. Higher positive correlation is depicted with darker color in Figure 1. The minimal correlation coefficient is 0.003 (between age and atrial fibrillation) whereas the maximal correlation coefficient is 0.29 (body-mass-index and diabetes mellitus) and thus the independent variables seem not to interact.

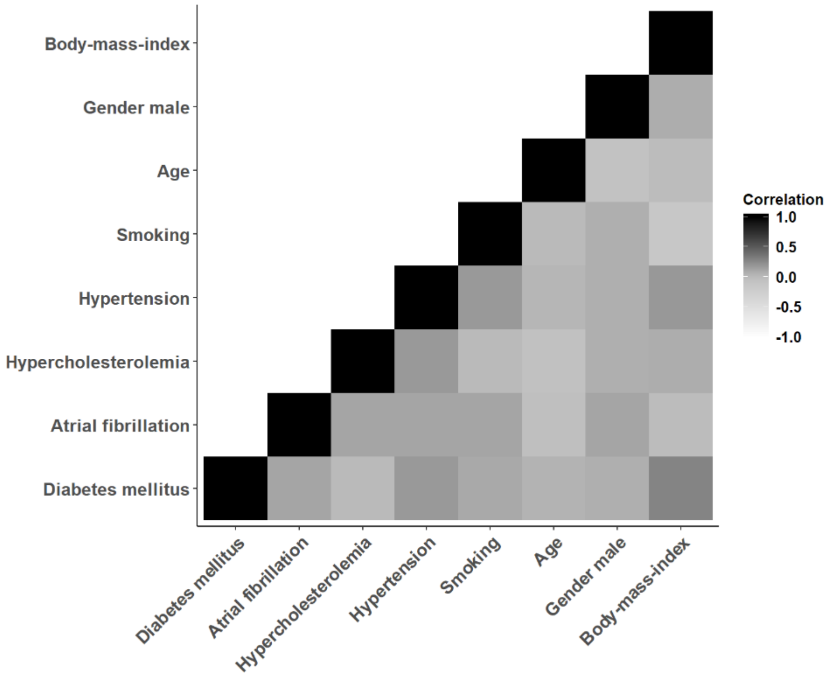

Figure 1. Correlation between independent variables. Higher positive correlation is depicted with darker color.

Further Results: Analyzing risk factors using the lower or mid interval time points for the event stroke

Table 1 shows the estimated weighted hazard ratio with 95% confidence intervals (CI) using the lower or mid interval time points for the event stroke and the weights 0.7 for “stroke” and 1 for “death”.

Table 1. Weighted Estimated Hazard Ratio using other time points.

	Weighted Hazard Ratio (95% CI) Using lower interval time	Weighted Hazard Ratio (95% CI) Using mid interval time
**Diabetes mellitus**	0.38 (0.20, 0.85)	0.39 (0.20, 0.86)
**Atrial fibrillation**	0.82 (0.40, 2.23)	0.82 (0.40, 2.23)
**Hypercholesterolemia**	1.26 (0.54, 2.49)	1.26 (0.54, 2.50)
**Hypertension**	1.22 (0.33, 2.68)	1.22 (0.34, 2.68)
**Smoking**	1.62 (0.84, 3.61)	1.61 (0.84, 3.58)
**Age**	1.16 (0.92, 1.66)	1.16 (0.92, 1.66)
**Male sex**	1.34 (0.65, 2.59	1.34 (0.65, 2.57)
